# Fortification of puffed biscuits with chitin and crayfish shell: Effect on physicochemical property and starch digestion

**DOI:** 10.3389/fnut.2023.1107488

**Published:** 2023-03-14

**Authors:** Chan Bai, Jiguo Zhu, Guangquan Xiong, Wenqing Wang, Juguang Wang, Liang Qiu, Qingfang Zhang, Tao Liao

**Affiliations:** ^1^Institute of Agro-Products Processing and Nuclear Agricultural Technology, Hubei Academy of Agricultural Sciences, Wuhan, China; ^2^Key Laboratory of Cold Chain Logistics Technology for Agro-Product, Ministry of Agriculture and Rural Affairs, Beijing, China; ^3^School of Petrochemical Science, Lanzhou University of Technology, Lanzhou, China

**Keywords:** chitin, crayfish shell, biscuits, pasting, starch digestion, physicochemical

## Abstract

Chitin is a polysaccharide and possesses numerous beneficial properties such as nontoxicity, biodegradability and biocompatibility, which draws much attention to its applications in food. Crayfish shell is a source of chitin alongside an antioxidants and a potential source of beneficial dietary fiber. In this study, chitin (CH) and crayfish shell (CS) with different concentrations were used to study their impact on pasting characteristics of flour mixture (wheat flour and glutinous rice flour) and influence on physicochemical and starch digestion property of puffed biscuit. The Rapid Visco-Analyzer results showed that the viscosity of powder mixture was decreased with the ratio of CH and CS increased. CH resulted in lowest peak viscosity and breakdown values of mixed powder. It was indicated that increasing amounts of CH and CS led to significantly reduced moisture content, expansion ratio but raised density of biscuits. CH and CS inhibited starch digestion and promoted a remarkable increase (*P* < 0.05) of resistant starch (RS) content. The hydrolysis kinetic analysis suggested a decelerating influence of CH on the hydrolysis content with lower values of equilibrium hydrolysis percentage (C_∞_) while CS on hydrolysis rate with lower kinetic constant (K). The estimated glycemic index (eGI) of the CH (15-20%) samples were below 55. These results are of great significance in delaying starch digestion and provided a better choice in design of fried puffed snacks for special crowd with chronic diseases such as diabetes, cardiovascular disease, and obesity.

## Introduction

Fried puffed foods are popular with consumers globally mainly due to their unique flavor, crispness, color, attractive taste and affordable cost (1). The main raw materials of the puffed food is digestible carbohydrates (starches) which are rapidly broken down within the human gastrointestinal tract. Different processing methods lead to different digestive property of starch. The frying process leads to the starch being more susceptible to enzymatic hydrolysis in gastrointestinal, thereby accelerating the starch conversion to sugar and causing a boost of postprandial blood glucose. Long-term hyperglycemia can cause chronic damage to various tissues and contribute to many human health problems, such as diabetes, high glycemic index, heart disease and obesity etc. (2). Over 415 million adults are suffering from diabetes globally and 318 million adults have impaired glucose regulation (3). Thereby, developing new methods to control the digestion rate of starch and maintain the stability of blood glucose level has become a key technological problem affecting human health. Starch could be classified into rapidly digestible starch (RDS), slowly digestible starch (SDS), and resistant starch (RS) based on its digestibility (4). Slowly digestible starch (SDS) and resistant starch (RS) can help control and prevent diseases such as diabetes and obesity (5). Therefore, it is important to design functional foods that can decrease the starch digestion and increase the SDS and RS fraction to maintain the glucose level in the blood. Nowadays, physical methods, enzymatic methods, and chemical method are main methods to inhibit starch digestion. However, problems as consumer safety, environmental pollution and efficiency have limited their application. Therefore, to develop a simple and efficient method to reduce the digestion of starch remains a challenge.

Dietary fiber, consisting of indigestible cellulose, hemicellulose, lignin, chitin, gums etc, are indigestible polysaccharides which is resistant to digestion in the small intestine and fermentation in the large intestine (6). In recent years, dietary fiber has gained roar attention worldwide as a promising functional food for improving physiological actions and regulating the health conditions of humans due to its potential function in decreasing cholesterol and blood glucose and prevention of some diseases, such as obesity, heart disease and atherosclerosis (7, 8). It was found that the digestibility, texture, sensory evaluation of starch based puffed snacks could be improved with the addition of dietary fiber as inulin and guar gum (9), etc. The dietary fiber particles dispersed within the starch matrix and crowded around the starch granules to inhibit the starch digestion.

Chitin, a dietary fiber, is a linear chain polymer composed of repeating units of β-(1 → 4)-2-acetamido-2-deowy-β-D-glucose, which has antioxidant, antidiabetic, lipid-lowering and antibacterial effects (10–12). It is safe and non-toxic, with good degradability and biocompatibility. The unique properties of chitin has made it being used amply in food industries and was thought as the third generation of health food. Recently, there has been great interest in using chitin or its derivatives as functional food ingredients (13). Zhou et al. (14) found that nanochitin could be used to retard lipid digestion in foods. Ji et al. (15) studied the impact of chitin on the gelatinization and retrogradation behaviors of starch. Chitin was also suggested as a potential novel form of prebiotics (16). Crayfish (*Procambarus clarkii*) has become common food source and approximately 100,000 tons of crayfish shells were generated every year, with about 80% of crayfish shells having been discarded as waste (17). The crayfish shell is an important natural source of chitin, chitosan and carotenoids. By-products like crayfish shell is increasingly explored as alternative food sources, due to its high nutritional value, cost-effective production and low carbon footprint (18). Consumption of chitin and crayfish shell was also correlated with a reduction in plasma TNF-α levels (16).

Although many efforts have been made to investigate the applications of chitin or chitosan in starch-based products (15), from the nutritional perspective, few studies have addressed the potential impact of chitin and crayfish shell on starch digestive fate in the human alimentary canal. There is still a relatively poor study of the interaction between chitin or crayfish shell with starch in puffed products. The frying process improved digestion of starch in gastrointestinal tract and led to increase in blood glucose levels. We hypothesized that the incorporation of chitin and crayfish shell could reorder the starch during frying process and bind around the starch, thereby inhibiting the digestion of starch by blocking amylase, thus further reduce glycemic index. Understanding the role of chitin and crayfish shell interactions on physicochemical properties and starch digestibility in biscuits may have important healthy benefits.

The purpose of our study was to develop an efficient method to control starch digestion in fried biscuit and made low glycemic index (GI) food by incorporating chitin and crayfish shell into flour mixture. The chitin and crayfish shell inhibited the starch digestion by forming barrier to prevent contact between starch and amylase. Chitin-biscuits (CH-biscuits) and crayfish shell-biscuits (CS-biscuits) containing chitin and crayfish shell at different ratios were prepared. The pasting, physicochemical property and morphology of biscuits was studied by scanning electron microscopy (SEM), texture profile analysis (TPA) and rapid visco analyzer (RVA). The *in vitro* starch digestibility of biscuits was also investigated. The research are of significance in inhibiting starch digestion and maintaining the health of special crowd with chronic diseases such as diabetes, cardiovascular disease, and obesity.

## Materials and methods

### Raw materials and chemicals

The chitin (CH) from crayfish shell was purchased from Shanghai Macklin Biochemical Co., Ltd. (Shanghai, China). The crayfish shell (CS) was kindly supplied by Wuhan Liangzihu Aquatic Processing Co. Ltd. (Wuhan, China). The α-amylase from porcine pancreas (4 u/mg) and Glycosylase (100,000 u/ml) were from Shanghai Yuanye Biotechnology Co., Ltd. (Shanghai, China). The low gluten wheat flour was purchased from Wuhan Suntyh Food Co., Ltd. and the glutinous rice flour was from Henan Enmiao Food Co., Ltd. Other ingredients including soybean oil, baking soda, yeast, salt and eggs were all available at local supermarket. All chemical reagents were of analytical grade purchased from Sigma-Aldrich (Germany).

The crayfish shells were washed thoroughly with distilled water to remove impurities, dried in a hot air oven at 105°C for 24 h, and pulverized to powder in a ultrafine pulverizer. After pulverizing, the crayfish shell powder was filtered through a 25 μm sieve to obtain a uniform powder and kept in a desiccator until application. Proximate composition of CS was determined in accordance with the methods in AOAC (19) and Ruth et al. (20). The CS contained 14.48 g of protein, 7.69 g of water, 0.2 g of fat, 28 g of chitin and 48.26 g of calcium salt (every 100 g).

### Preparation of samples

The procedure for making the biscuits was followed the method of AACC (21). Firstly, the flour mixture (wheat flour and glutinous rice flour) were mixed in a ratio of 11:3, to which, different levels 5, 10, 15 and 20% of CH or CS were added, respectively (based on the amount of wheat flour and glutinous rice flour) and mixed well. Then, 0.7% of yeast powder, 3% of salt, 3% of baking soda, 67% of egg and 10% of water were added to the mixture and mixed in a dough mixer for 10 min to form dough. The dough was fermented in an incubator at 37°C for 3 h and then the fermented dough was rolled and cut into 0.6 cm × 0.5 cm × 3 cm biscuit flans. The biscuits were fried in a soybean oil pan at 190 ± 10°C for 50 s and removed to a tray, drained the oil, cooled at ambient temperature, packed in polyethylene bags and stored at 20°C for further analysis.

### Pasting properties

A Rapid Visco Analyzer (RVA-TM, Perten Instruments, Sweden) was used to measure the pasting characteristics of the mixed powder (wheat flour and glutinous rice flour with different content of CH and CS) according to the method of Feng et al. (22) with slightly modified. Firstly, the flour mixture slurry (14%, 3 g total weight) was suspended in 25 mL denized water in RVA container. Then the CH or CS (0, 5, 10, 15, and 20% of flour mixture; w/w) were added, respectively. The calculated amounts of CH or CS powder were pre-stirred for 30 s by plastic paddle. The detection procedure was set as: at the beginning of 10 s, the paddle speed was 960 rpm, then the paddle speed slowed down to 160 rpm and kept until the end of the detection. The temperature was set as: held at initial temperature 50°C for 1 min, then heated to 95°C in 4 min, held at 95°C for 25 min, at last cooled to 50°C within 4 min and held at 50°C for 2 min. all the measurements were performed in triplicate and the averages were reported.

### Physicochemical properties

#### Expansion ration and volume density

The volume of biscuit was determined using a seed displacement method. The bulk density was calculated by dividing its determined volume by the mass of biscuit (23). The expansion ration was calculated by dividing its volume after puffing by volume before puffing of biscuit (24). The expression was as follows:


(1) $$E\,x\,p\,a\,n\,s\,i\,o\,n\,r\,a\,t\,i\,o\,n=\frac{v\,o\,l\,u\,m\,e\,a\,f\,t\,e\,r\,p\,u\,f\,f\,i\,n\,g}{v\,o\,l\,u\,m\,e\,b\,e\,f\,o\,r\,e\,p\,u\,f\,f\,i\,n\,g}$$

#### Moisture content and oil content

The moisture content of the samples was calculated based on the weight difference measured before and after drying the samples in a hot air oven at 105°C for 24 h (25). The oil content of the samples was obtained as follows: The samples were dried at 105°C to a constant mass, and they were placed into petroleum ether (60–80°C) as solvent to measure the oil absorbed by the samples with a Soxhlet extractor (SOX405 purchased from Hanon Instrument Co., Ltd.). Oil content was expressed as a percentage of total oil content on a dry weight basis. Analysis was subject to three samples.

#### Water solution index (WSI) and water absorption index (WAI)

Both WSI and WAI were defined by Kowalski et al. (26). 1 g of grated sample was suspended in 15 mL water at room temperature for 30 min, stirred gently during this process and then centrifuged at 3,000 x *g* for 15 min. The supernatant was then poured into a pan of known weight. WSI refers to the weight of dried solids in the supernatant, expressed as a percentage of the original weight of the sample. WAI refers to the weight of gel obtained by removing the supernatant from the original dried solid of unit weight. Such measurement was subject to three samples. The expressions of WAI and WSI were as follows:


(2) $$W\,A\,I\,\left(g/g\right)=\frac{\mathrm{weight}\,\mathrm{of}\,\mathrm{sediment}}{\mathrm{sample}\,\mathrm{dry}\,\mathrm{weight}}$$

(3) $$W\,S\,I\,\left(g/k\,g\right)=\frac{\mathrm{weight}\,\mathrm{of}\,\mathrm{dry}\,\mathrm{dish}-\mathrm{weight}\,\mathrm{of}\,\mathrm{empty}\,\mathrm{dish}}{\mathrm{sample}\,\mathrm{dry}\,\mathrm{weight}}$$

#### Textural properties

The textural characteristics of biscuits were measured with the help of a TA-XTPlus Texture analyzer (Stable Micro System, Vienna court, UK) with a P36/R aluminum cylindrical probe. The parameters were set as: pretest speed 1.0 mm/s, test speed 0.5 mm/s, post-test speed 1.0 mm/s, trigger force 5.0 g, and deformation level 75%. The harness and springiness was obtained from the force-time curve of the texture profile, respectively.

#### Scanning electron microscope

The surface morphology of CH-biscuits and CS-biscuits at different magnification (× 100 and × 30) were observed using a scanning electron microscope (MIRA4, TESCAN Ltd., Bmo, Czech) with an accelerating voltage of 2 kV. The freeze-dried biscuits were adhered to brass slip under argon environment by a gold sputter module in a high vacuum evaporator to form a gold layer with thickness of 20 nm.

#### Sensory evaluation

Twenty well-trained assessors (16 females and 4 males, 20–30 years) evaluated the finished CH-based biscuits and CS-based biscuits for their appearance, color, taste, texture and overall acceptability. The trained sensory panel were all from Institute of Agro-Products Processing and Nuclear-Agricultural Technology (Wuhan, China). A nine-point preference scale was used to evaluate the overall acceptability for determining sensory attributes of the above samples (From point 1-9, where 1 is strongly dislike, 5 is acceptable and 9 is strongly like) (27). The biscuits samples were placed on a panel, and each assessor was asked to observe and taste individually.

The sensory evaluation experiment was conducted according to the guidelines of the Declaration of Helsinkiwas. All work with human subjects performed here was reviewed and approved by the Hubei Academy of Agricultural Sciences Institutional Review Board (IRB). The products tested were safe for consumption.

#### *In vitro* digestion of starch in CH-biscuits and CS-biscuits

The *in vitro* starch digestion of CH and CS-based biscuits samples was determined based on the modified method of Zhang et al. (28). 3 g of pancreatic α-amylase (14 u/mg)was dissolved in 10 mL sodium acetate buffer (0.1 M, pH = 5.2) and centrifuged at 3000 × g for 5 min. The supernatant was collected and mixed with 1.3 mL of amyloglucosidase (100,000 u/ml) in a beaker. The samples to be tested were prepared so that they all contained the same initial quantity of starch (60.54 g/100 g). The biscuits samples (100 mg, dry basis) were added into 25 mL sodium acetate buffer (0.1 M, pH = 5.2) and then they were equilibrated at 37°C. Then 1.5 mL of enzyme solution (pancreatic α-amylase and amyloglucosidase) were added and incubated for 180 min at 37 °C with continuous agitation (180 rpm). At 5, 10, 20, 30, 60, 90, 120, 150, and 180 min, aliquots (0.1 mL) were removed and mixed with 8.0 mL of 80% ethanol to inhibit the enzymes. Afterward, the mixed solution was centrifuged at 5000 rpm for 20 min, and the glucose content in the supernatant was measured using a 3,5-dinitrosalicylic acid (DNS) method (29). Rapidly digestible starch (RDS), slowly digestible starch (SDS) and resistant starch (RS) were calculated as follows:


(4) $$RDS\left(\%\right)=\frac{G_{20}\times 0.9}{\text{TS}}\times 100\%$$

(5) $$SDS\left(\%\right)=\frac{\left(G_{120}-G_{20}\right)\times 0.9}{\text{TS}}\times 100\%$$

(6) RS(%)=1-(RDS+SDS)

Where: G_20_ and G_120_ represent the glucose contents produced of hydrolysis within 20 min and 120 min, respectively, and TS is the total starch content of the sample (initial amount of starch). A factor 0.9 indicated the conversion factor of glucose into starch.

The rate of starch digestion kinetics were estimated based on the approach described elsewhere (30). The starch hydrolysis can be fitted to a first-order eqution,


(7) $$C_{t}={\text{C}}_{\infty }\times \left(1-e^{-k\,t}\right)$$

Where: Ct (%) is the percentage of starch digested at t time (0, 5, 10, 20, 30, 60, 90, 120, 150, 180 min); C∞ is the estimated percentage of starch digested at 180 min; K(min^–1^) is the starch digestion rate constant. The areas under hydrolysis curves (AUC, 0-180 min) were calculated as the integral of the kinetic equation and used to obtain the HI (Hydrolysis Index). The HI was calculated by dividing the AUC of the samples by that of white bread (as a reference). The following formula proposed by Goñi et al. (31) was used to calculate the eGI (estimated glycemic index) value.


(8) eGI=39.71+0.549⁢HI

### Data analysis

All experiments were repeated three times. All data were expressed as the mean ± standard deviation (SD). Experimental data were analyzed using analysis of variance (ANOVA), expressed as the mean value ± SD, and significant differences among means were determined by Duncan’s test. All analyses were performed using IBM SPSS Statistics 25 (SPSS Inc., Chicago, IL). The significance level in all cases was set at *P* < 0.05. All the analysis and data visualizations were conducted on the Origin Pro 2018.

## Results and discussion

### Pasting properties of base material

**FIGURE 1:**
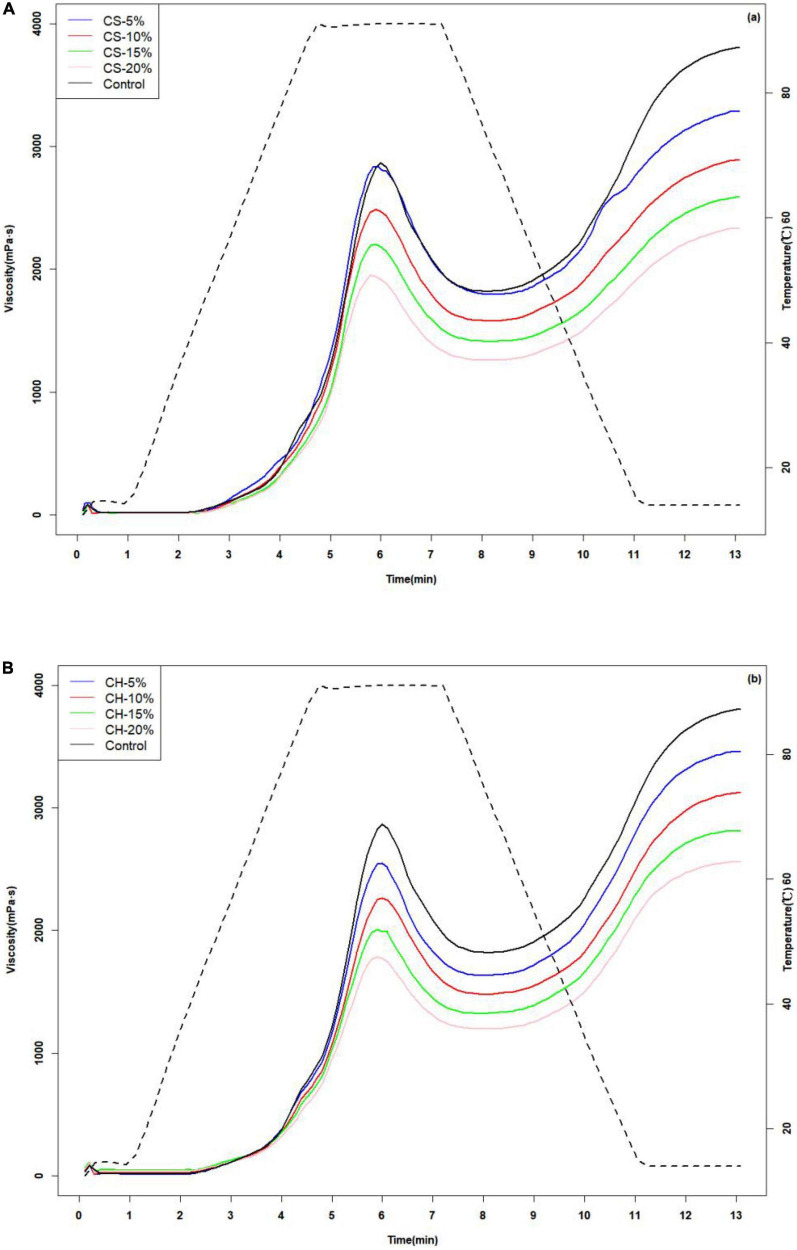
Viscosity changes of CS-flour mixtures at different mixing ratios (CS/flour mixture = 0, 5, 10, 15, and 20% w/w) **(A)** and CH-flour mixtures at different mixing ratios (CH/flour mixture = 0, 5, 10, 15, and 20% w/w) **(B)**.

The effect of CH and CS on the pasting properties of flour mixture was showed in Figures 1A, B. The pasting curve was often used to describe the swelling and disruption of starch granules in the aqueous phase. CH-flour and CS-flour mixtures were differed in the pasting behavior, which were observed much lower viscosity during heating and cooling. The effect was dependent on the CH (and CS) concentration. As shown in Figure 1A, CS5% mixed with flour did not have significant impact on the viscosity of flour during the heating step, while the CS10-20% decreased the viscosity. It suggested that the high concentration of CS played a role in starch swelling in the initial stage of pasting. Table 1 showed the parameters that define the pasting behavior of CH-flour and CS-flour mixtures with different addition ratios of CH or CS (CH or CS/flour mixture = 0, 5, 10, 15, and 20%, w/w).

**TABLE 1:** Pasting properties of mixed powder with different contents of CH and CS.

Sample		Peak viscosity/mPa⋅s	Trough viscosity/mPa⋅s	Final viscosity/ mPa⋅s	Breakdown/mPa⋅s	Setback/mPa⋅s	Pasting temperature/°C
Control	0%	2879.1 ± 19.8^f^	1837.5 ± 17.77^h^	3831.5 ± 41.72^h^	1041.5 ± 2.12^e^	1994.3 ± 24.04^h^	82.25 ± 0.5^ab^
CS	5%	2846.5 ± 12.02^f^	1810.2 ± 15.56^h^	3287.1 ± 0.14^f^	1036.5 ± 3.54^e^	1477.2 ± 15.56^e^	74.70 ± 0.35^ab^
	10%	2503 ± 19.8^e^	1590.5 ± 13.43^f^	2901.6 ± 12.73^d^	912.5 ± 6.36^d^	1310.5 ± 0.71^c^	81.85 ± 0.57^a^
	15%	2208.5 ± 6.36^d^	1417.5 ± 7.78^d^	2593.2 ± 0.21^b^	791.3 ± 1.41^c^	1175.5 ± 7.78^b^	83.95 ± 0.07^d^
	20%	1960 ± 12.73^b^	1266.5 ± 7.78^b^	2343.5 ± 8.49^a^	693.5 ± 4.95^b^	1076.5 ± 0.71^a^	83.95 ± 0.04^d^
CH	5%	2535.5 ± 26.16^e^	1626.5 ± 12.02^g^	3452.2 ± 16.97^g^	909.3 ± 14.14^d^	1825.5 ± 4.95^g^	82.23 ± 0.07^ab^
	10%	2249.5 ± 24.75^d^	1474.5 ± 6.36^e^	3109.5 ± 24.04^e^	775 ± 18.38^c^	1634.5 ± 17.68^f^	82.78 ± 0.6^bc^
	15%	2007.5 ± 7.78^c^	1317.5 ± 12.73^c^	2815.5 ± 7.78^c^	690.5 ± 4.95^b^	1498.5 ± 4.95^e^	83.21 ± 0.2^cd^
	20%	1787.2 ± 9.9^a^	1192.3 ± 5.66^a^	2564.5 ± 0.71^b^	595.2 ± 4.24^a^	1372.5 ± 4.95^d^	83.58 ± 0.84^cd^

Values are mean ± standard deviation of three replicates. Means with different letters within each column are significantly different (*P* < 0.05, Ducan’s test). CH (5, 10, 15, and 20%) were CH-flour mixtures at different mixing ratios (CH/flour mixture = 5, 10, 15, and 20% w/w); CS (5, 10, 15, and 20%) were CS-flour mixtures at different mixing ratios (CS/flour mixture = 5, 10, 15, and 20% w/w); Control was flour mixture; CH indicates chitin; CS indicates crayfish shell.

Increasing amount of CH and CS caused a significant declining trend of peak viscosity, trough viscosity and final viscosity (*P* < 0.05). The addition of CH led to a significant decrease in peak viscosity from 2879 mPa⋅s (control) to 1787 mPa⋅s (CH20%). This could be attributed to the fact that chitin interacted with wheat starch and glutinous rice starch granules through hydrogen bonding, inhibiting the swelling of the granules and leading to lower peak viscosity. The experimental results of Liu et al. (32) also showed that polysaccharides could inhibit the expansion of starch granules, leading to the decrease of viscosity. As to CS20% group, a reduction of peak viscosity about 31.9% when compared with control was observed, which might because of the addition of crayfish shell powder contributing to a dilution of the starch granules concentration in the system thus decreasing the peak viscosity (33). The final viscosity of CH group was also decreased, which might be due to the interaction between CH and starch amylose molecules. It was indicated that modifications which result from the addition of chitin to a starch system were complex, and these could be ascribed to polymers interactions or phase separation processes. We proposed the chitin be around the starch and form a starch-chitin network structure by hydrogen bonds. The starch-chitin structure was also been reported in other literature (34, 35). An increase in CH content led to re-crystallization of dispersed amylose chains, which retarded short-term retrogradation of starch and decreased final viscosity (36).

The increasing addition ratio ranging from 5%∼20% of CH or 10% ∼20% CS both caused a significant decrease of breakdown viscosity (*P* < 0.05). The smaller breakdown viscosity indicated that the structure of starch granules during starch gelatinization was more stable and the damage of starch granules by heating and shear force was reduced (32). As both CH and CS could significantly reduce the breakdown viscosity (*P* < 0.05), the addition of CH and CS made the starch granules with more stable structures. System viscosity was mainly affected by three factors: granules swelling degree; granules disruption and the surface interaction especially the system was added with polysaccharide, which would influence the amylose leaching out the swollen granules (37, 38). High amount of CH or CS could wrap around the starch, inhibit the swelling content of granules and impede the dissipate of leached amylose. Yuris et al. (39) have found that wheat starch mixed with mesona chinensis polysaccharide also showed decreased pasting viscosity.

The setback of mixed powder decreased from 1,994 mPa⋅s to 1,372.5 (CH20%) and 1,076.5 mPa⋅s (CS20%) with the content of CH (CS) increased from 0 to 20%. Setback viscosity was an index of starch retrogradation, demonstrating the trend of starch paste to retrograde. The decrease in the setback viscosity implied a prevention of short-term retrogradation. It might be explained that –OH groups of CH interacted with the –OH groups of wheat and glutinous starches to form a hydrogen bridge of CH-amylose, thus reducing the association of amylose molecules and inhibiting the amylose rearrangement (32) Qin et al. (40) found that the addition of chitin nanowhiskers into starch products could delay the short-term and long-term aging of starch, which was consistent with the results in this paper.

### Physicochemical characteristics of biscuits

#### Expansion ration and density

CH and CS both had significant effects on the puffing degree and density of biscuits (*P* < 0.05) (Table 2). The density was inversely correlated with expansion ration of biscuits. When the addition of CH and CS were 20%, the puffing degree decreased by 24.14 and 43.84%, respectively, while the density increased by 39% and 23.21% when compared with the control. The reason might be that chitin was a rigid material with fibrous nature and high mechanical strength, by which it could cause the low adhesion between starch and CH (and CS) and the weakened interaction between starch molecules (41). The molten starch stick to the chitin wall and formed a complex wall that hindered the expansion. The rearrangement of starch molecules during frying were affected by CH and the aging time of starch were delayed, resulting in increased density and reduced ability for expansion of puffed products (42). The results were in agreement with the research found from Jiamjariyatam et al. (43). The puffing degree of CS group was less than that of CH group.

**TABLE 2:** Physicochemical properties of fried biscuits with different contents of CS and CH.

Sample		Puffing degree	Density (g/cm^3^)	Moisture (%)	Oil (%)	WAI (g/kg)	WSI (g/g)	Hardness (kg)	Springiness
Control	0%	2.03 ± 0.03^d^	0.56 ± 0.02^ab^	2.07 ± 0.06^c^	21.34 ± 0.48^a^	373.1 ± 3.35^e^	8.34 ± 0.17^a^	8.83 ± 0.2^a^	79.73 ± 0.23^g^
CS	5%	1.83 ± 0.05^cd^	0.59 ± 0.05^abc^	1.23 ± 0.07^b^	22.43 ± 0.63^ab^	322.32 ± 0.69^d^	8.94 ± 0.74^ab^	15.27 ± 0.23^b^	69.29 ± 1.11^ef^
	10%	1.64 ± 0.03^abc^	0.58 ± 0.01^abc^	0.98 ± 0.07^ab^	23.06 ± 0.81^ab^	302.97 ± 2.35^b^	9.11 ± 0.09^ab^	20.52 ± 0.14^c^	50.59 ± 0.3^b^
	15%	1.61 ± 0.04^abc^	0.62 ± 0.02^bcd^	0.7 ± 0.04^ab^	26.01 ± 0.62^cd^	301.17 ± 1.78^b^	9.42 ± 0.2^ab^	22.51 ± 0.87^d^	44.74 ± 0.74^a^
	20%	1.41 ± 0.03^a^	0.69 ± 0.04^de^	0.45 ± 0.09^a^	27.19 ± 0.80^d^	280.13 ± 1.51^a^	11.58 ± 0.46^c^	26.51 ± 0.36^e^	55.00 ± 0.71^c^
CH	5%	1.87 ± 0.18^cd^	0.55 ± 0.04^a^	0.95 ± 0.05^ab^	21.59 ± 2.72^a^	366.12 ± 4.44^e^	9.93 ± 1.65^b^	14.22 ± 0.79^b^	69.94 ± 0.54^f^
	10%	1.72 ± 0.2^bc^	0.62 ± 0.04^abcd^	0.67 ± 0.09^ab^	21.92 ± 0.26^ab^	320.58 ± 2.20^cd^	13.13 ± 0.8^d^	23.51 ± 0.46^d^	68.24 ± 0.59^e^
	15%	1.66 ± 0.24^abc^	0.64 ± 0.05^cd^	0.47 ± 0.01^a^	22.18 ± 1.34^ab^	315.77 ± 9.85^cd^	14.69 ± 0.36^e^	27.37 ± 0.7^e^	60.71 ± 0.27^d^
	20%	1.54 ± 0.12^ab^	0.67 ± 0.02^d^	0.46 ± 0.03^a^	24.18 ± 1.14^bc^	313.40 ± 1.77^c^	14.92 ± 0.14^e^	39.08 ± 0.69^f^	60.91 ± 0.93^d^

Values are mean ± standard deviation of three replicates. Means with different letters within each column are significantly different (*P* < 0.05, Ducan’s test). CS (5, 10, 15, and 20%) were biscuits with different contents of chitin (CS/flour mixture = 5, 10, 15, and 20% w/w); CH (5, 10, 15, and 20%) were biscuits with different contents of crayfish shell (CH/flour mixture = 5, 10, 15, 20% w/w); Control were biscuits without the addition of any CH or CS; CH indicates chitin; CS indicates crayfish shell.

#### Moisture content and oil content

According to Table 2, The moisture content and oil content were negatively correlated. When 20% of CH and CS were added, moisture content of both group decreased by 1.62%, while oil content increased by 2.84 and 5.85% when compared with the control. That was because the strong hydrogen bonds in chitin and between chitin nanowhiskers and starch molecules therefor caused strong hydrophobicity of CH (40), so the moisture in the product cannot be firmly locked during the process of frying. On the other hand, lower moisture content showed higher heat absorption of the food during process. The high temperature during the frying process resulted in oxidation of the products and different types of derivative were produced after the break down of lipid compounds, whose heat transfer efficiency was significantly lower than that of lipid molecules. Chitin has strong antioxidant activity and strong protective effect on lipid oxidation, which could significantly reduce the production of oil derivative, hence improve the heat transfer efficiency of oil and decrease the moisture content (44). The effect of CS on oil content of biscuits was significantly higher than that of CH, that was because besides of chitin, CS also contained a large amount of proteins. The proteins and protein hydrolysates may lead to reduced surface tension and increased hydrophobicity, which facilitated the entrance of oil, enhancing the oil content of biscuit. Although the crayfish shell and chitin both could increase the oil content when added into biscuits, the oil content of obtained CH or CS-biscuits was < 24 g/100 g, which is less than that in commercial fried (35 g/100 g) and fiber-fortified fried potato snacks (30.6-33.2 g/100 g) (45).

#### Water absorption index (WAI) and water soluble index (WSI)

Water absorption index (WAI) is a measure of the water-holding capacity of the starch, cellulose and protein in products after swelling in excessive water (46). It is the demonstration of the behavior of puffed product during its interaction with water and the degree of the starch conversion (47). WAI was also used to indirectly evaluate the porosity of material. Water-soluble index (WSI) is a measure of the degradation of starch molecules during processing which increased the amount of soluble polysaccharide. WSI reflects the solubility of product components in water (48). As shown in Table 2, WAI decreased with the addition of CH and CS, but the WSI was opposite. In 20% CH and 20% CS, WAI decreased by 16 and 24.92%, while WSI increased by 78.90 and 38.85% compared with the control, respectively. The increasing CH and CS content led to decreased WAI, which might because CH and CS affected the starch molecular. The availability of hydrophilic group influences the WAI (49). The addition of CH and CS may decrease the extent of starch gelatinization during frying and cause reduced water absorption. Similarly, Singh et al. (50) reported a decrease in WAI with addition of pea grits in extrusion of rice due to the dilution of starch in rice pea blends. WSI increased as the increase of CH and CS content. WSI was a parameter that reflected the degradation suffered by the components of the biscuits. Chitin molecules disrupted continuous structure of the melt in frying and impeded elastic deformation. It could improve the heat transfer efficiency of oil which making the internal temperature of biscuits rise faster, resulting in the increase of water-soluble substances. Kowalski et al. (26) also reported a increased in WSI with the raising temperature. The WSI of CH group was significantly higher than that of CS group (*P* < 0.05) due to higher chitin quantity.

#### Textural properties of biscuits

The textural changes of puffed biscuits fortified with different levels of chitin or crayfish shell are presented in Table 2. The hardness values increased significantly (*P* < 0.05) with a corresponding increase in CH or CS concentration. In 20% CH and 20% CS, the hardness reached 39.08 ± 0.69 kg and 26.51 ± 0.36 kg. The hardness value correlated with density, expansion and thickness of cell walls (51). Puffed biscuits enriched with high level of chitin showed high hardness values, which was because of their compact, hard, not so crunchy nature owing to their low expansion properties. The addition of chitin powder contributed to premature rupture of gas cells which reduced the expansion and the porosity of puffed biscuits (52). As with CS-biscuits, except for chitin, the harder texture of the biscuits was attributed to the increased protein and calcium content and their interaction during dough development and frying (53). Texture profile analysis revealed significant differences (P < 0.05) in springiness characteristics between control and different treatments. A decreasing trend of springiness was observed with increasing CH or CS levels. Low springiness reflects the tendency to crumble upon external forces (54). CH and CS exerted negligible effects on the hardness and springiness, indicating unacceptable texture with higher levels of CH or CS inclusion in biscuits.

#### Surface morphology of biscuits

**FIGURE 2:**
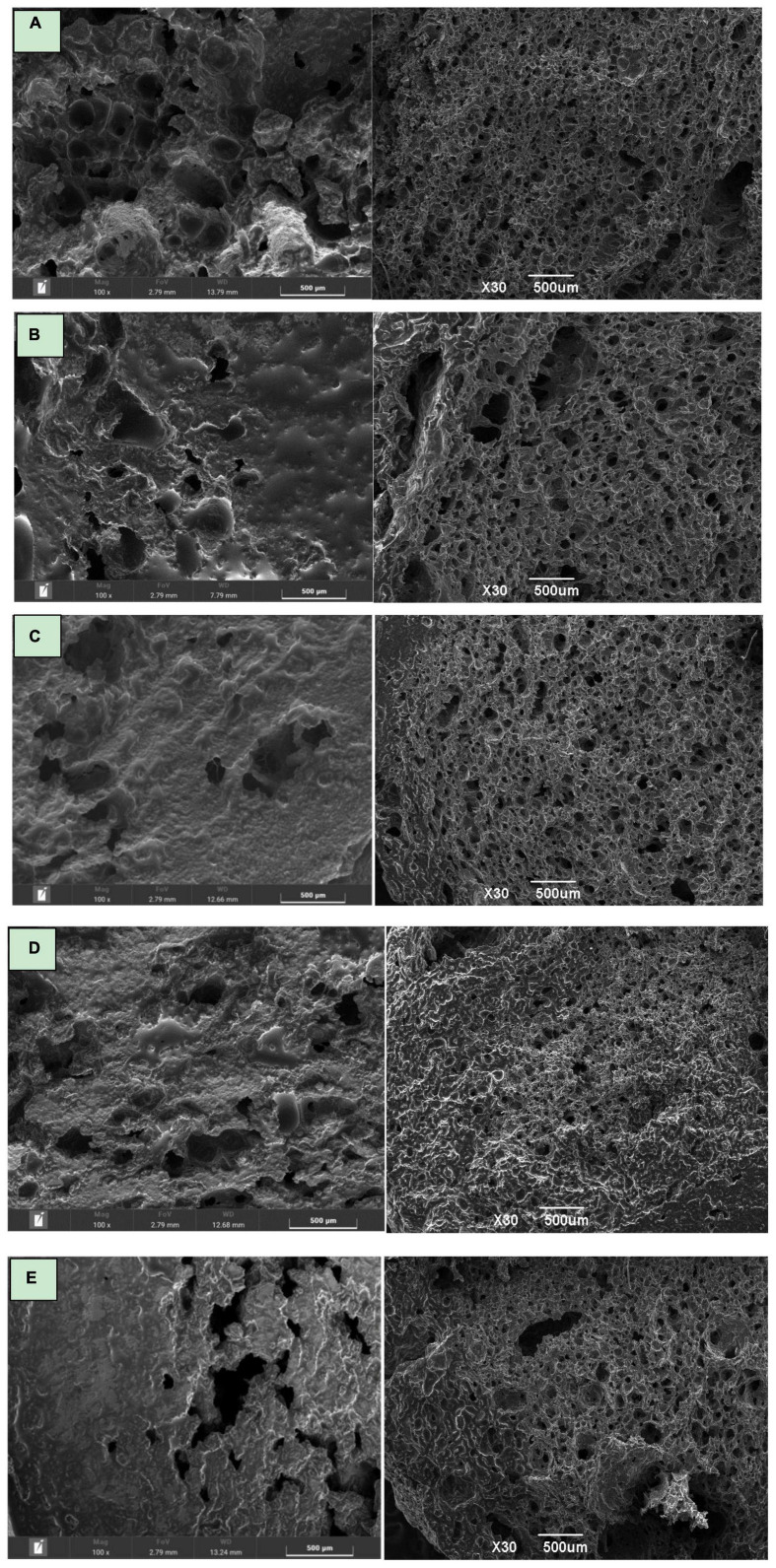
Scanning electron microscope at different magnification (× 100 and × 30) of CH-biscuits and CS-biscuits with different additions (CH or CS/flour = 0, 10% and 20%); **(A)** control sample, **(B)** 10% CS- biscuits, **(C)** 20% CS-biscuits, **(D)** 10% CH-biscuits, **(E)** 20% CH-biscuits; CH indicates chitin; CS indicates crayfish shell.

Scanning electron microscope (SEM) of the cross section of the fried biscuits were showed in Figure 2, from which the organizational structure of the biscuits could be clearly seen. In Figure 2A, the surface of control sample presented a porous spongy structure, with ellipse and thick-shaped pores distributed evenly. As compared to the control, when the addition were 10% and 20% of CS and CH as showed in Figures 2B–E, it could be observed that the number of pores was significantly reduced with irregular shape and uneven distribution, showing an overall less expansion of fried puffed products than control sample. Figures 2B, C showed smaller cell size and thicker cell walls with respect to the Figure 2A, since the CH 10% and CS 10% contained chitin and crayfish shell powder fragments. Chitin fibrils in starch powder resulted in premature rupture of gas cells which reduces porosity and expansion of the functional snacks. The Figures 2C, E indicated that the structure of less expanded products was more closeness and the surface was smoother. According to Figure 2E, in CH 20% group, instead of being puffed and porous, the biscuits split unevenly. It might because that too much addition of CH could result in reduced binding capacity of dough thus decreased the viscoelasticity. At the same time, uneven heating of biscuit interior with CH during frying could also lead to the surface morphology.

#### Sensory characteristics of biscuits

**FIGURE 3:**
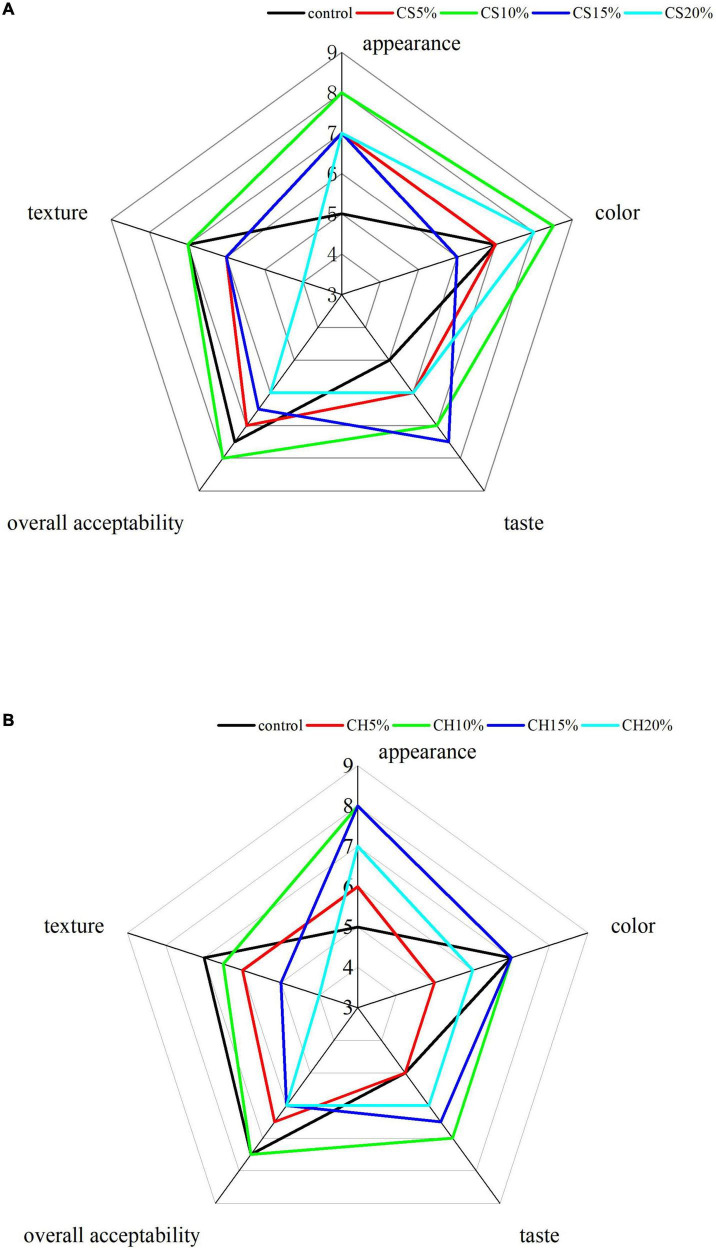
The sensory properties of **(A)** biscuits with different additions of CS and **(B)** biscuits with different additions of CH. CS 5, CS 10, CS 15, and CS 20% were CS-biscuits at different addition ratio (CS/flour = 5, 10, 15, and 20%); CH 5, CH 10, CH 15, and CH 20% were CH-biscuits at different addition ratio (CH/flour = 5, 10, 15, and 20%); CH indicates chitin; CS indicates crayfish shell.

The incorporation of either CH or CS at different addition ratios (0, 5, 10, 15 and 20%) on judging scores of sensory quality characteristics: appearance, color, taste, texture and overall acceptability of produced biscuits were studied and the results were shown in Figure 3. For CH group, when 10% of CH was added, the appearance, color and taste were highest, scoring 8, 7 and 7, respectively, and the overall acceptability scored top out at 7.5. The CS 10% group was preferred to the control and scored slightly higher than the control especially for color, appearance and overall acceptability, the overall acceptability scoring top out at 8. On the other hand, CH or CS 15∼20% group exhibited a reduction in judging scores, especially for texture and taste due to less expansion ration the chitin. From the present results for sensory evaluation of the fried biscuits, it could be concluded that the CH and the CS should be incorporated into the biscuits up to the incorporation level of 10% from each.

#### *In vitro* digestion studies

**FIGURE 4:**
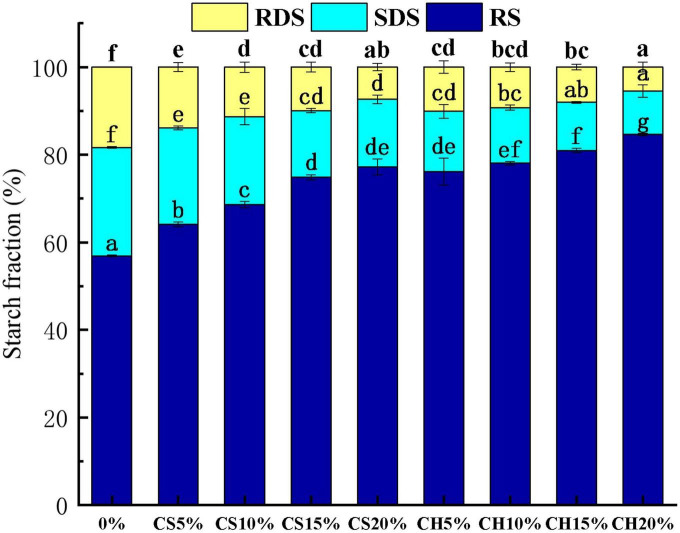
The percentages of RDS, SDS and RS content of biscuits with different addition ratio of CS and CH. RDS, fast digestion starch; SDS, slow digestion starch; RS, resistant starch; CS 5, CS 10, CS 15, and CS 20% were CS-biscuits at different addition ratio (CS/flour = 5, 10, 15, and 20%); CH 5%, CH 10%, CH 15% and CH 20% were CH-biscuits at different addition ratio (CH/flour = 5, 10, 15, and 20%); CH indicates chitin; CS indicates crayfish shell.

The digestion property is an important property of starch-based samples. The RDS, SDS, and RS contents of control, CH-biscuits and CS-biscuits were shown in Figure 4. CH and CS significantly influenced the starch digestible property and the influence was related to the addition ratio of CH and CS. It could be obviously observed that with the increasing of CS, the RS content of CS-biscuit increased, but the RDS and SDS contents decreased significantly. The CH-biscuits showed higher RS and lower SDS content when compared with the CS-biscuits. The CH20% group exhibited the highest RS (84.6%) content and lowest RDS (9.92%). The possible reason was that CH and CS entangled with starch to form integrated network structure and increased the complexity of starch composition, which caused an increase in RS content (55). The formed CS or CH-starch structure enhanced the resistant starch content. We proposed that a layer of polysaccharide around the surface of starch granules to prevent amylase from digesting starch. The chitin and crayfish shell around starch had a barrier effect on starch, and this barrier effect increased as the concentration of chitin and crayfish shell increased. Low concentration of chitin formed a weak chitin-starch network, which was insufficient to block amylase. As the concentration of chitin increased, the protective effect of the chitin on starch granules was enhanced. The chitin prevent the binding between enzyme and starch so as to slow down the hydrolysis rate of starch (56). Wang et al. (57) revealed chitin nanowhiskers could bind with pepsin in simulated gastric fluid due to hydrogen bonding and van der Waals forces, leading to the aggregation and alteration of micro-environment of aromatic amino acids in pepsin. Similar phenomenon was also observed by other studies (58). With the same addition amount, RS of CH group were higher than that of CS group, which indicated that the effect of CH on starch in biscuits was more significant than CS (*P* < 0.05).

#### Kinetics of starch hydrolysis

**FIGURE 5:**
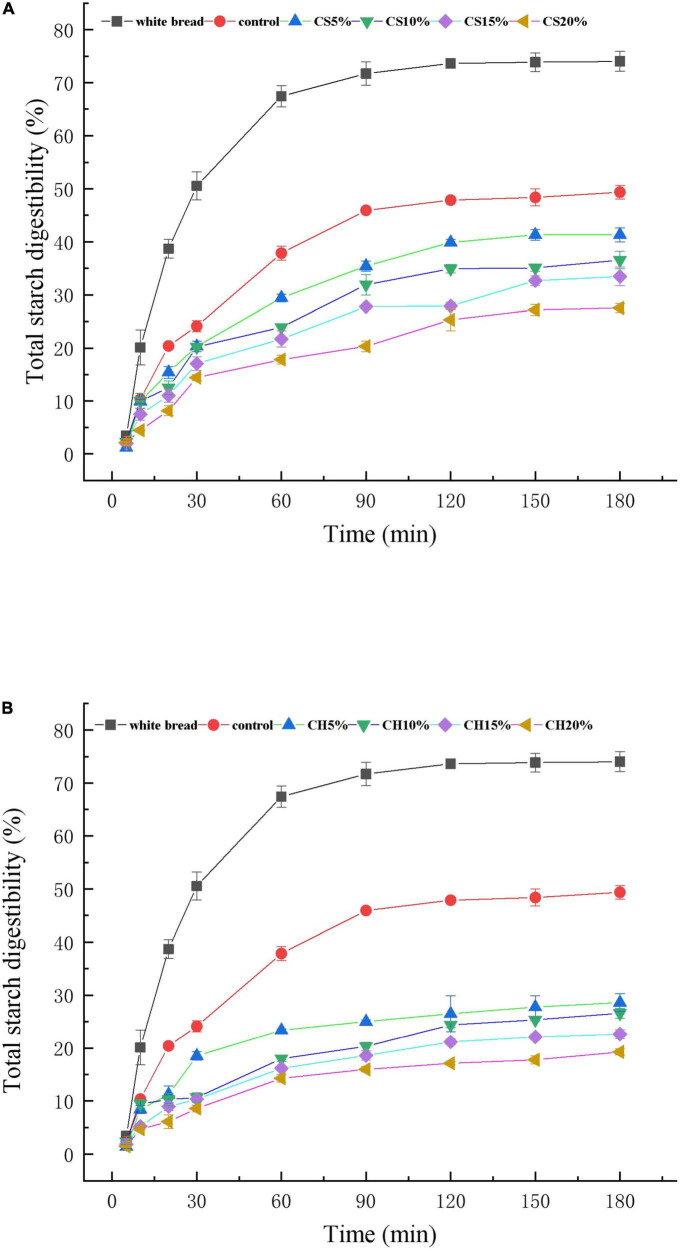
Impact of CS **(A)** or CH **(B)** on the *in vitro* hydrolysis profiles of starch in CH-biscuits and CS-biscuits at different additions (CS or CH/flour = 5, 10, 15, and 20%); White bread as a reference; Error bars represent standard deviation from the mean of triplicate measurements.

The digestion of the various biscuits samples (white bread, control, CH 5%∼20% and CS 5%∼20%) was measured using an *in vitro* method based on the same starch content in Figure 5. All the samples followed the same trend for starch hydrolysis, which rose rapidly within 0∼20 min, then increased slowly after 60 min and gradually reached equilibrium after 120 min. The CH-biscuits, which contained different amounts of chitin, displayed the lowest degree of hydrolysis. At the final of digestion, the starch hydrolysis degree of CH-biscuits and CS-biscuits were 19.24 ± 0.60% and 27.53 ± 0.83%, respectively, much lower than the control (49.36 ± 1.27%). The addition of CH resulted in a decrease in the starch hydrolysis, which could be attributed to the increase of hydrophobicity. Moisture content also played an important role in the hydrolysis of starch. The hydrophobic nature of chitin might limit the availability of water for enzyme substrate reactions, reducing the overall hydrolysis of starch and hydrolysis kinetics. On the other hand, the chitin around the starch granules could limit access of enzymes to starch, leading to the decrease of enzymatic starch hydrolysis. CH-biscuits had significantly lower digestibility than CS-biscuits during the whole digestion period.

To further evaluate the effect of CH and CS on digestible properties of starch, the maximum starch hydrolysis degree (C_∞_), the kinetic constant (K), and the estimated glycemic index (eGI) of each of CH-biscuits and CS-biscuits samples were calculated using a fitting model that assumes first-order kinetics (Eq. 5) to the data (Table 3). The C_∞_ values were estimated concentration of hydrolyzed starch, which was related to the equilibrium concentration of the digestion. The C_∞_ values were decreased from 50.67 to 28.5 and 19.04%, with the addition of CS or CH from 0 to 20%. The K value, which was relevant to the reaction rate of starch hydrolysis, was different between the CH-biscuits and CS-biscuits. The K value of the CS-biscuits ranged from 0.0223 to 0.0177 min^–1^, which was lower than CH-biscuits samples (from 0.0301 to 0.0211 min^–1^). The results suggested that the addition of CH decreased the extent of starch digestibility, while the CS decreased the rate of starch digestibility. The nature of chitin determines its physico-chemical behavior and this may affect the rate of digestion of carbohydrates and absorption of sugars in the small intestine. The estimated glycemic index (eGI) of biscuits decreased gradually with the increasing addition of CH and CS, and significant difference (*P* < 0.05) was observed between the eGI of the CH and CS groups. It has been reported that eGI above 70 is classified as high blood glucose food, 55 to 69 as middle blood glucose food, and below 55 as low blood glucose food (59). The 20% CH group had the lowest eGI (52.00 ± 0.48) and could be owned to low eGI food. All the CS-biscuits and CH-biscuits with lesser CH addition were belong to middle blood glucose food. Therefore, the addition of CH and CS could affect the digestion properties of starch in biscuits and reduce the eGI value. CH laid a greater effect on reducing starch digestion in biscuits than CS, resulting in the eGI of CH group lower than CS group. That was because the CH effected in retarding starch digestion and eGI through either formation of a physical barrier or alteration of the starch granule crystal structure, both decreasing the extent of starch digestibility and the rate of starch digestibility. The CS contain protein, calcium, ash, etc. The CS acted as physical barrier which mainly decreased the rate of starch digestibility. Considering that low glycemic food are desirable to generate and moderate postprandial glucose and insulin response, the CH-biscuits with higher content of CH would be advisable.

**TABLE 3:** Digestion parameters obtained from the digestibility kinetics of CH-biscuits and CS-biscuits.

Biscuit	Addition amount	C_∞_ (%)	K (min^–1^)	HI	eGI
Control	0%	50.67 ± 1.31^g^	0.0232 ± 0.0012^b^	61.31 ± 2.16^g^	73.37 ± 0.20^g^
CS	5%	42.62 ± 0.78^f^	0.021 ± 0.0007^ab^	50.04 ± 1.12^f^	67.18 ± 0.62^f^
	10%	36.68 ± 0.17^e^	0.0223 ± 0.0021^ab^	43.87 ± 0.12^e^	63.80 ± 0.06^e^
	15%	33.30 ± 1.9^d^	0.0203 ± 0.0033^ab^	38.67 ± 0.78^d^	60.94 ± 0.43^d^
	20%	28.5 ± 0.71^c^	0.0177 ± 0.002^a^	31.55 ± 0.84^c^	57.03 ± 0.46^c^
CH	5%	27.91 ± 1.92^c^	0.0301 ± 0.0025^c^	36.07 ± 0.79^d^	59.51 ± 0.43^d^
	10%	26.24 ± 0.60^c^	0.0220 ± 0.0014^ab^	30.71 ± 0.25^c^	56.57 ± 0.13^c^
	15%	22.89 ± 0.66^b^	0.0212 ± 0.0025^ab^	26.96 ± 0.58^b^	54.51 ± 0.32^b^
	20%	19.04 ± 0.09^a^	0.0211 ± 0.0022^ab^	22.39 ± 0.87^a^	52.00 ± 0.48^a^

Values are expressed as mean ± standard deviation of three replicates. Means with the different superscript letters within each column are significantly different (*P* < 0.05, Ducan’s test). C_∞_, equilibrium constant; K, kinetic constant; HI, hydrolysis index; eGI, estimated glycemic index. CS (5, 10, 15, and 20%) were biscuits with different contents of chitin (CS/flour mixture = 5, 10, 15, and 20 w/w); CH (5, 10, 15, and 20%) were biscuits with different contents of crayfish shell (CH/flour mixture = 5, 10, 15, and 20% w/w); Control were biscuits without the addition of any CH or CS; CH indicates chitin; CS indicates crayfish shell.

## Conclusion

The chitin and crayfish shell had significant effects on the physicochemical properties of biscuits and the digestion properties of starch. The moisture content and expansion ratio of the biscuits decreased with the increasing amount of CH and CS, while the oil content and density showed the opposite. *In vitro* digestion simulation test showed that CH and CS could reduce the percentage of ready digestible starch (RDS) and slow digestible starch (SDS) by 28 and 20% while increasing the resistant starch (RS). Both CH and CS could slow down starch hydrolysis of biscuits. It was indicated that the structure formed by chitin and crayfish shell around the starch surface, which provided protection to the starch granules, prevented the entry of amylase, and slowed starch digestion. The protecting also restricted the swelling of the starch granules. The addition of CH and CS to hinder starch digestion in fried biscuits had healthy consequences, which indicated that CH-biscuits and CS-biscuits were belong to middle eGI food, even some were low eGI food. The CH and CS may help to lower the risk of high blood glucose levels caused by fried biscuits and maintain sensory quality at same time. The research results are of great significance in delaying starch digestion. However, the oil content of the fried biscuits was also a potential danger for health, and future research should identify methods of simultaneously reduce the oil content and the digestibility of starch.

## Data availability statement

The original contributions presented in this study are included in the article/supplementary material, further inquiries can be directed to the corresponding authors.

## Author contributions

CB: conceptualization, verification, draft writing, and project administration. JZ: review and editing and supervision. GX: data curation, validation, and formal analysis. WW: data collection, data interpretation, validation, and language polish. JW: investigation, validation, and data curation. LQ: methodology and validation. QZ: project administration and conceptualization. TL: editing, methodology, resources, and funding acquisition. All authors contributed to the article and approved the submitted version.
